# Overexpression of constitutively active mitogen activated protein kinase kinase 6 enhances tolerance to salt stress in rice

**DOI:** 10.1186/1939-8433-6-25

**Published:** 2013-10-28

**Authors:** Kundan Kumar, Alok Krishna Sinha

**Affiliations:** National Institute of Plant Genome Research, Aruna Asaf Ali Road, New Delhi, 110067 India; Birla Institute of Technology & Science Pilani, K K Birla Goa Campus, Zuarinagar, Goa 403726 India

**Keywords:** MAPK, MAPKK, Rice, Salt stress, Transgenic

## Abstract

**Background:**

Salinity is one of the most common abiotic stresses encountered by plants in the environment and transgenic approaches offer new opportunities to improve tolerance. The mitogen activated protein kinase (MAPK) kinase (MKK) is a key component of MAPK cascade that plays important roles in intra and extra cellular signaling in plants. In the present study, a MKK from rice (*Oryza sativa)*, OsMKK6 was functionally characterized in salt stress by transforming its constitutively active form.

**Findings:**

*OsMKK6* was made constitutively active by mutating serine and threonine to glutamic acid by site directed mutagenesis, and transformed in indica cultivar rice var. Pusa Basmati-1. The transgenic seedlings growing in 200 mM NaCl solution showed increased root/shoot length and weight, less chlorophyll beaching and higher MAPK activity compared to the wild types.

**Conclusion:**

Present work suggest role of *OsMKK6* gene in salt stress signaling in rice.

**Electronic supplementary material:**

The online version of this article (doi:10.1186/1939-8433-6-25) contains supplementary material, which is available to authorized users.

## Findings

The MAPK cascade function significantly in salt or osmotic stress signaling that has been well established in yeast and human cells (Jonak et al. 2002). Rice genome analysis revealed the presence 15 MAPKs, 8 MKKs and 75 MKKKs (MAPK group, 2002Sinha et al. 2011Kumar et al. 2013). Out of eight MAPKKs reported from rice, partial functional characterization of only *OsMKK6* has been carried out so far. OsMKK6 (earlier named as OsMEK1) specifically and physically interacted with OsMPK3 (Wen et al. 2002Xie et al. 2012) in moderate low temperature stress signaling. In our present study, we established the functional role of OsMKK6 in salinity stress by transgenic approach. A constitutively active form of OsMKK6 was generated and transformed in Pusa Basmati-1 (PB1). Our results suggest that overexpression of constitutively active form of OsMKK6 enhance tolerance to salt stress in rice.

### Salt stress regulates expression of *OsMKK6*

**Figure 1:**
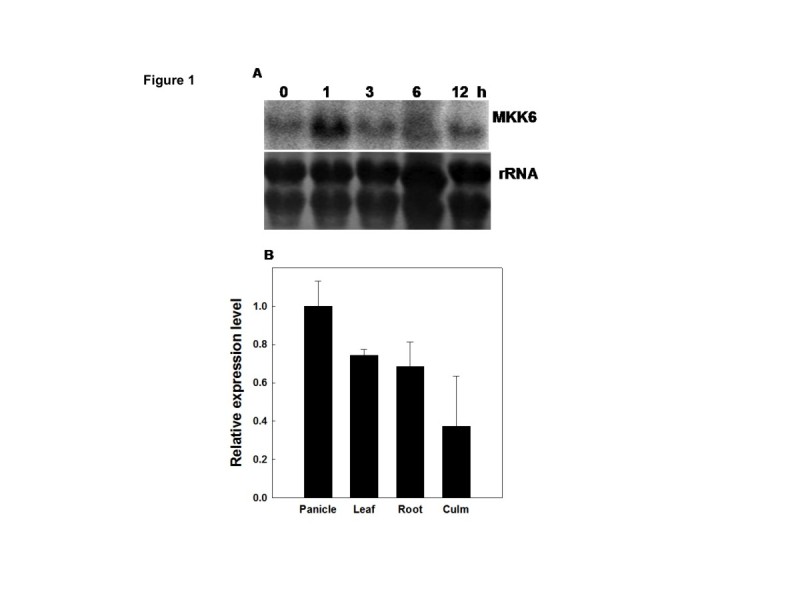
**Expression profile of** ***OsMKK6.*** **(A)** Northern blot analysis of *OsMKK6* from four week old rice leaves. 20 μg of RNA was loaded in each lane. The experiment was repeated three times. Lower panel shows equal loading, rRNA stained with methylene blue. **(B)** Tissue-specific expression of *OsMKK6* in rice tissues. Samples of the leaf, culm, panicle and roots of 3 months old rice plant were taken and frozen. Expression of the *OsMKK6* in different tissues, detected by qRT–PCR with the rice actin as a control. SD from three independent experiment has been represented. The relative mRNA level was calculated taking the transcript in the panicle as 1.

We reported earlier the differential transcript regulation of *OsMKKs* by abiotic stresses and that *OsMKK6* transcript gets upregulated after 1 hour of salt stress (Kumar et al. 2008). Northern blot analysis on RNA isolated from rice seedling exposed to salt stress (200 mM NaCl) at different time points showed a transient increase of *OsMKK6* transcripts within one hour of salt stress (Figure 1A). Among tissue specific expression *OsMKK6* showed highest expression level in panicle followed by leaves, roots and least in culms (Figure 1B) (primer details please see Additional file 1: Table S1).

The analysis of *OsMKK6* expression profile in ssp japonica was studied by employing massively parallel signature sequencing (MPSS) database using twenty nucleotide signature tags (Brenner et al. 2000). *OsMKK6* showed more transcript accumulation in both roots and leaves in salt stress whereas in cold stress transcript accumulation was higher in roots but showed less accumulation of transcript in drought stressed leaves (Additional file 2: Figure S1A). Transcript of *OsMKK6* was noticed in almost all the tissue and its accumulation was highest in mature roots (Additional file 2: Figure S1B). OsMKK6 expression level remain highest in overall tissues and the stresses, as analyzed by MPSS suggesting it as one of the key member of MKK, being integral part of almost all the MAPK cascade and plays an important function in integrating upstream signals for appropriate cellular responses.

### Generation of constitutive active OsMKK6 transgenic lines

Dominant mutants of constitutively active form of protein kinases are useful for determining various protein functions. MAPKK proteins can be mutated to constitutively active form by substituting acidic residues for both of serine (S) or threonine (T) residues in the consensus sequence (Ren et al. 2002). Gain of function alleles of MKK2 were generated by changing both putative phosphorylation site to glutamic acid (E) residue (T220E and T226E) (Teige et al. 2004).

To investigate the functional role of *OsMKK6* the constitutively active OsMKK6^EE^ was generated by changing the putative phosphorylation sites from S and T to E residue (S221E and T227E) by site directed mutagenesis (Primer listed in Additional file 1: Table S1). The full length OsMKK6^EE^ cDNA was cloned in binary construct pCAMBIA 1303 under the control of constitutive 35S CaMV promoter and transformed into *Agrobacterium* cells and subsequently used for rice transformation (Additional file 3: Figure S2A-H). To confirm the presence of *OsMKK6* in transgenic lines, genomic PCR analysis was carried out using end sequence of CaMV 35S promoter as forward primer and end sequence of MKK6 gene as reverse primer (Additional file 4: Figure S3A). Expression level of independent transgenic lines E1, E2, E7, E8, E9, E10, E13, E16, E17 and E18 were analysed by RNA gel blot analysis. All transgenic lines except E8, showed higher steady state level of OsMKK6 in overexpressed transgenic line compared to wild type (WT) and E10, E13, E18 transgenic lines showing higher transcript level were used for stress tolerance studies (Additional file 4: Figure S3B). The stable inheritance of transgenes of the transformed plants was evaluated by resistance to hygromycin in T_1_ generation. Homozygous lines (E10, E13 and E18) were selected according to germination percent (100%) in ½ MS with hygromycin (50 mg/l) in the T_2_ generation after 10 days.

Irrespective of growth stage, transgenic rice plants developed normally and showed no growth retardation. To analyze the physiological competence of transgenic plants, chlorophyll fluorescence was measured in 5 overexpressed transgenic lines as well as three WT PB1 rice plant. The Fv:Fm value for all the plants were between 0.73 to 0.76 indicating that there is no change in photosynthetic efficiency. The effective quantum yield (yield II) of transgenic lines were also similar with that of WT (Additional file 5: Figure S4) indicating non significant change of photosynthesis in overexpressed transgenic lines as compared to WT.

### Constitutively active OsMKK6 overexpressed lines exhibited higher salt stress tolerance

**Figure 2:**
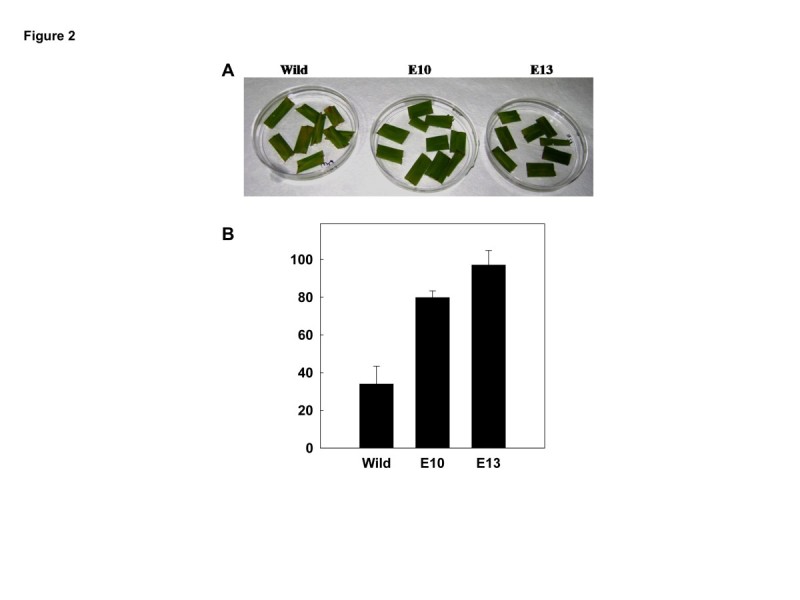
**Analyzing salt tolerance of OsMKK6**^**EE**^**overexpressed transgenic plants. (A)** Leaf disc assay, the phenotypic appearance of the transgenic leaf segments as compared to wild type (control) leaves. **(B)** Percentage chlorophyll retention of transgenic leaves. The asterisks represent the significant difference in cholorphyll retention compared with wild type (WT) plants, (*) P < 0.05, (**) P < 0.01.

**Figure 3:**
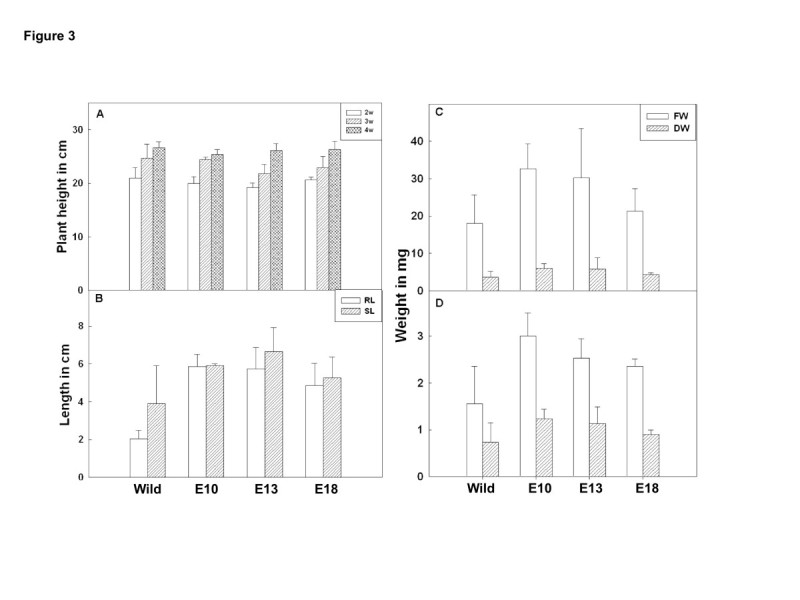
**Analysis of OsMKK6**^**EE**^**overexpressed transgenic line at seedling stage in salt stress. (A)** Plant height in centimeter of wild type and three transgenic lines after 2, 3 and 4th week of normal growth in green house. **(B)** Root and shoot length in cm after 2 weeks of growth in MS medium containing salt solution (200 mM NaCl). SD were calculated from 3 different biological samples. **(C)** Fresh weight (FW) and dry weight (DW) of wild type and three transgenic line rice seedlings in milligram after three weeks of growth in MS media containing salt solution (200 mM NaCl). **(D)** FW and DW of wild type and three transgenic line rice roots in milligram after three weeks of growth in MS media containing salt solution (200 mM NaCl). The asterisks represent the significant difference compared to control as wild type (WT) plants, (*) P < 0.05, (**) P < 0.01.

**Figure 4:**
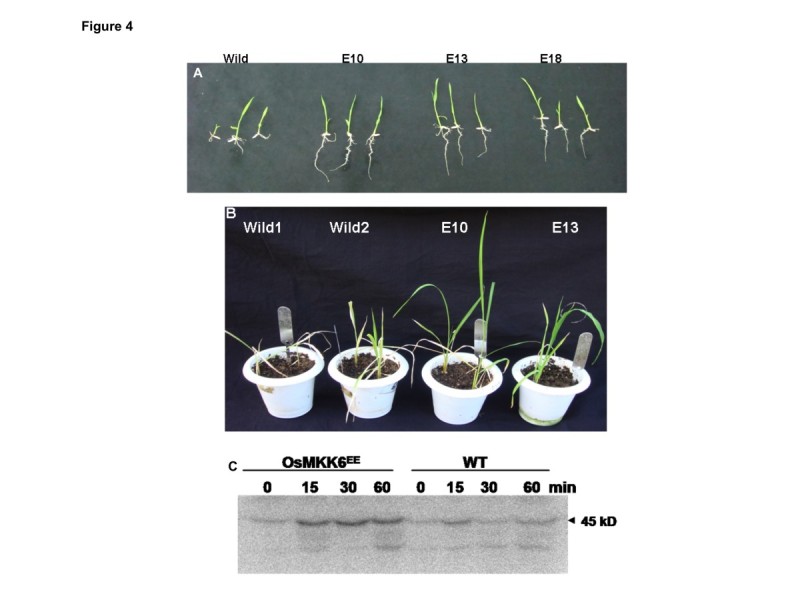
**OsMKK6**^**EE**^**overexpressed transgenic lines confers tolerance and higher MAPK activity. (A)** Two weeks old seedlings grown in MS media with 200 mM NaCl. **(B)** Salt stress were given by 2 weeks old plants soaked in 200 mM NaCl solution for 1 week and then supplied with fresh water for 2 more week for recovery. **(C)** In gel kinase assay of transgenic (*OsMKK6*^*EE*^) and wild type (WT) plants in salt solution (200 mM NaCl) and harvesting the samples at different time points. Salt stress was given by dipping the rice plant in Hoagland solution containing 200 mM NaCl solution.

Leaf disc assay to show salinity stress tolerance in rice was performed according to Kanneganti et al. (2008) by floating leaf discs on NaCl for 72 h and estimating chlorophyll retention. Transgenic leaf segment of E10 and E13 line could retain ~80% and ~95% of chlorophyll respectively in contrast to the leaf segment from the WT which retained only 32% of chlorophyll (Figure 2A, B). The damage caused by stress was reflected in the degree of bleaching in the leaf tissue. Plant height of both WT and transgenic rice plants recorded at seedling stage showed no significant difference between them (Figure 3A). For salt stress tolerance studies, the protocol described by Xiang et al. (2007) was followed. Surface sterilized seeds of both WT and positive transgenic lines (selected by germinating the seeds on MS medium containing 50 mg/L hygromycin) were grown on MS agar supplemented with 200 mM NaCl, and observed their performance under stress. All the transgenic grew well, and their shoots and root length recorded were significantly longer than WT after growing for 2 weeks (Figure 3B). In comparison to WT, transgenic lines accumulated significantly higher fresh and dry weight of shoots and roots in salinity stress (Figure 3C, D). At germination stage, transgenic rice seeds overexpressing OsMKK6^EE^ were placed in media containing MS supplemented with 200 mM NaCl. Overexpressed OsMKK6^EE^ seedling showed better phenotypes in terms of roots and shoots compared to WT (Figure 4A). To analyze stress at plantlet stage, 2 week old transgenic rice plants grown in the pots were subjected to salt stress with 200 mM NaCl for two week and then subsequently watered the same plants with fresh water for another two weeks for recovery. Salt stress severely inhibited growth of shoots of WT compared to three independent transgenic plants (Figure 4B).

To show constitutive expression of MKK6 in transgenic plants, in-gel kinase activity was performed using MBP as substrate under salt stress. Salt stress in transgenic and WT rice plants were given by dipping plant in Hoagland solution containing 200 mM NaCl solution and harvested at different time points. In salt stress higher MBP phosphorylation activity was observed at 44 kDa. Transgenic rice plant harbouring MKK6^EE^ showed higher phosphorylation activity at 19 kDa after 15 minutes of salt stress (Figure 4C). Earlier studies also demonstrated the activation of MAPKs in the plants exposed to salinity (Munnik et al. 1999).

Previously, it has been proved that modulating MKKs in plants results in higher abiotic stress tolerance. In *Arabidopsis* overexpression of constitutively active MKK2 showed enhanced freezing and salt tolerance (Teige et al. 2004). The mkk9 mutant of *Arabidopsis* is salt insensitive and germinated on media containing 150 mM NaCl (Alzwiy and Morris 2007). AtMEK1 overexpressed plants were more tolerant to drought and salt stresses (Xing et al. 2007). Expression of active MKK9 protein enhances the sensitivity of transgenic seedlings to salt stress, while loss of MKK9 activity resulted in reduced salt sensitivity (Xu et al. 2008). Similarly in this report, we provided evidence for the role of MKK6 in salt stress signaling by overexpression studies of constitutive active form of OsMKK6 in rice.

In conclusion, this study has provided insight into the role of OsMKK6 in salinity stress in rice. The transgenic seedlings grown in 200 mM NaCl solution showed enhanced root, shoot length, weight and MBP phosphorylation activity compared to WT. Inactivation of OsMKK6 using either T-DNA insertion mutants or RNAi approach will further clarify the role of OsMKK6 in the cascade involved in salt stress signaling and future studies will be focused in this direction.

## Electronic supplementary material

Additional file 1: Table S1: List of primers used in the study. (PDF 53 KB)

Additional file 2: Figure S1: Expression data of MKK6 in salinity, drought and cold stress of rice young root and young leaves library. Y axis show transcript per million (TPM) showing abundance of the gene in the library. NYR: Young roots; NSR: Young roots treated with 200 mM salt stress for 24 hours; NDR: Young roots treated with drought stress for 5 days; NCR: Young roots treated with cold stress (4°C) for 24 hours. NYL: Young leaves; NSL: Young leaves treated with 200 mM salt stress for 24 hours; NDL: Young leaves treated with drought stress for 5 days; NCL: Young leaves treated with cold stress (4°C) for 24 hours. (C) Tissue specific expression data of MKK6 of rice. NRA: Mature roots (60 days) replicate A; NGD: Germinating seedling grown in dark; NST: mature stem (60 days); NLA: mature leaves (60 days) replicate A; NME: crown vegetative meristematic tissue (60 days); NPO: mature pollen; NOS: ovary and mature stigma; NIP: immature panicle; NGA: germinating seeds; NCA: callus (35 days). (PDF 423 KB)

Additional file 3: Figure S2:*Agrobacterium* mediated transformation of rice (PB1). (A) T-DNA in binary vector pCAMBIA 1303 containing full length *OsMKK6* with mutation at S221E and T227 at *Nco* I/*Bgl* II sites. (B) Callus emerges from rice seeds on MS medium supplemented with 2,4-D. (C) calli ready for *Agrobacterium* transformation. (D) regenerating calli on selection media containing 50 mg/lt hygromycin. (E) Putative transgenic plants transferred to culture bottles on MS for rooting. (F) Rooted plants are transferred to pots in green house. (G) Mature plants after 3 months of growth. (H) Plants bearing panicles in green house after 5 months of regeneration. (PDF 531 KB)

Additional file 4: Figure S3: Confirmation of transgenic lines. (A) Testing of 39 putative transgenic lines for the presence of *OsMKK6* transgene by total genomic PCR using flanking sequence of pCAMBIA primer, ‘C’ is the amplification of control (wild type) and M is DNA ladder (500 bp). (B) Northern blot analysis of 10 transgenic lines overexpressing *OsMKK6*^*EE*^ lines. *OsMKK6* cDNA was used as a radiolabeled probe for northern hybridization, C is wild type rice plants. The lower panel show methylene blue stained rRNA for equal loading and RNA quality. (PDF 375 KB)

Additional file 5: Figure S4: Chlorophyll fluorescence of overexpressed lines. (A) Maximum quantum yield (Fv/Fm), (B) Effective quantum yield (Yield II) of overexpressed transgenic lines. SD from three different leaves with three points on one leaf of same line has been represented. No statistically significant difference was noticed with control as wild type 3 at P < 0.05 and P < 0.001. (PDF 333 KB)

Below are the links to the authors’ original submitted files for images.

Authors’ original file for figure 1

Authors’ original file for figure 2

Authors’ original file for figure 3

Authors’ original file for figure 4
