# Supplementary material for: Overexpression of constitutively active mitogen activated protein kinase kinase 6 enhances tolerance to salt stress in rice
Source: Rice (N Y). 2013 Oct 28;6:25. doi: 10.1186/1939-8433-6-25 (PMC4883705; doi:10.1186/1939-8433-6-25)
Supplement: Supplementary file 1 — Additional file 1: Table S1: List of primers used in the study. (PDF 53 KB) [file 12284_2013_76_MOESM1_ESM.pdf]

Table S1 List of primers used in the study.

S.No.	Genes	Purpose	Primer sequence
1	Actin	qRT-PCR	5′ CGGTGTGATGGTTGGTATGG 3′<br>5′ GCCTCAGTCAGCAACACAGG 3′
2	MKK6	qRT-PCR	5′ TCCGAGGAAACTGCAGATGA 3′<br>5′ TTTGCGAACTGCCTCTTGAA 3′
3	MKK6^{EE}	Constitutively active	5′ GCAAGTGAAATGGGTCAGCGGGATGAGTTTG 3′<br>5′ CAAACTCATCCCGCTGACCCATTTCACTTGC 3′
4	MKK6^{EE}	To clone MKK6^{EE} in pCAMBIA1303	5′ CATGCCATGGATGAGGGGGAAGAAGCCGCAC 3′<br>5′ GAAGATCTTTACTCGGATATATTCATTGGAG 3′
5	MKK6	To clone in pBSK for RNA gel blot	5′ GGAATTCCCATGAGGGGGAAGAAGCCGCACAAG 3′<br>5′ TCCCCCGGGGGATTACTCGGATATATTCATTGGAGGTTC 3′
6	CaMV MKK6rev	Genomic PCR	5′ CGATAAAGGAAAGGCCATCGTTGAA 3′<br>5′ TTACTCGGATATATTCATTGGAG 3′
