# Supplementary material for: Overexpression of constitutively active mitogen activated protein kinase kinase 6 enhances tolerance to salt stress in rice
Source: Rice (N Y). 2013 Oct 28;6:25. doi: 10.1186/1939-8433-6-25 (PMC4883705; doi:10.1186/1939-8433-6-25)
Supplement: Supplementary file 2 — Additional file 2: Figure S1: Expression data of MKK6 in salinity, drought and cold stress of rice young root and young leaves library. Y axis show transcript per million (TPM) showing abundance of the gene in the library. NYR: Young roots; NSR: Young roots treated with 200 mM salt stress for 24 hours; NDR: Young roots treated with drought stress for 5 days; NCR: Young roots treated with cold stress (4°C) for 24 hours. NYL: Young leaves; NSL: Young leaves treated with 200 mM salt stress for 24 hours; NDL: Young leaves treated with drought stress for 5 days; NCL: Young leaves treated with cold stress (4°C) for 24 hours. (C) Tissue specific expression data of MKK6 of rice. NRA: Mature roots (60 days) replicate A; NGD: Germinating seedling grown in dark; NST: mature stem (60 days); NLA: mature leaves (60 days) replicate A; NME: crown vegetative meristematic tissue (60 days); NPO: mature pollen; NOS: ovary and mature stigma; NIP: immature panicle; NGA: germinating seeds; NCA: callus (35 days). (PDF 423 KB) [file 12284_2013_76_MOESM2_ESM.pdf]

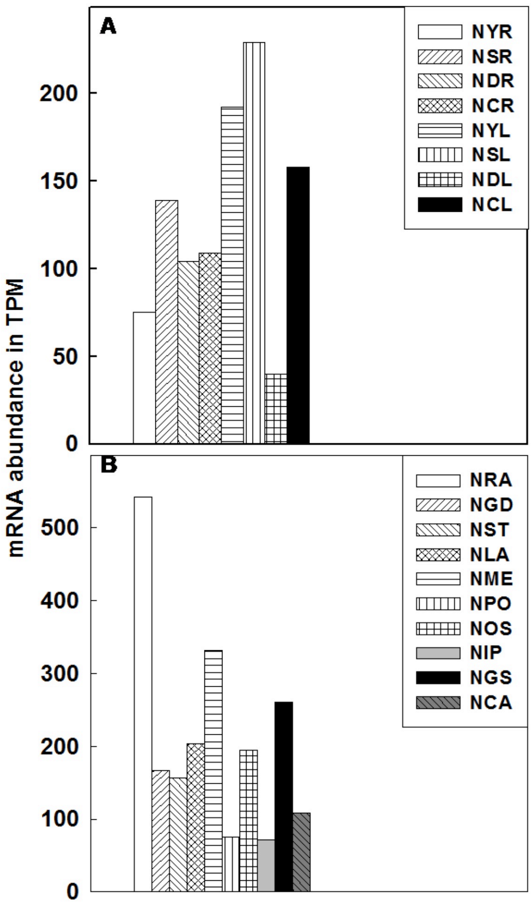

Figure S1 Expression data of MKK6 in salinity, drought and cold stress of rice young root and young leaves library. Yaxis show transcript per million (TPM) showing abundance of the gene in the library. NYR: Young roots; NSR: Young roots treated with 200mM salt stress for 24 hours; NDR: Young roots treated with drought stress for 5 days; NCR: Young roots treated with cold stress (4°C) for 24 hours. NYL: Young leaves; NSL: Young leaves treated with 200mM salt stress for 24 hours; NDL: Young leaves treated with drought stress for 5 days; NCL: Young leaves treated with cold stress (4°C) for 24 hours. (C) Tissue specific expression data of MKK6 of rice. NRA: Mature roots (60 days) replicate A; NGD: Germinating seedling grown in dark; NST: mature stem (60 days); NLA: mature leaves (60 days) replicate A; NME: crown vegetative meristematic tissue (60 days); NPO: mature pollen; NOS: ovary and

mature stigma; NIP: immature panicle; NGA: germinating seeds; NCA: callus (35 days).
