# Supplementary material for: Overexpression of constitutively active mitogen activated protein kinase kinase 6 enhances tolerance to salt stress in rice
Source: Rice (N Y). 2013 Oct 28;6:25. doi: 10.1186/1939-8433-6-25 (PMC4883705; doi:10.1186/1939-8433-6-25)
Supplement: Supplementary file 3 — Additional file 3: Figure S2:Agrobacterium mediated transformation of rice (PB1). (A) T-DNA in binary vector pCAMBIA 1303 containing full length OsMKK6 with mutation at S221E and T227 at Nco I/Bgl II sites. (B) Callus emerges from rice seeds on MS medium supplemented with 2,4-D. (C) calli ready for Agrobacterium transformation. (D) regenerating calli on selection media containing 50 mg/lt hygromycin. (E) Putative transgenic plants transferred to culture bottles on MS for rooting. (F) Rooted plants are transferred to pots in green house. (G) Mature plants after 3 months of growth. (H) Plants bearing panicles in green house after 5 months of regeneration. (PDF 531 KB) [file 12284_2013_76_MOESM3_ESM.pdf]

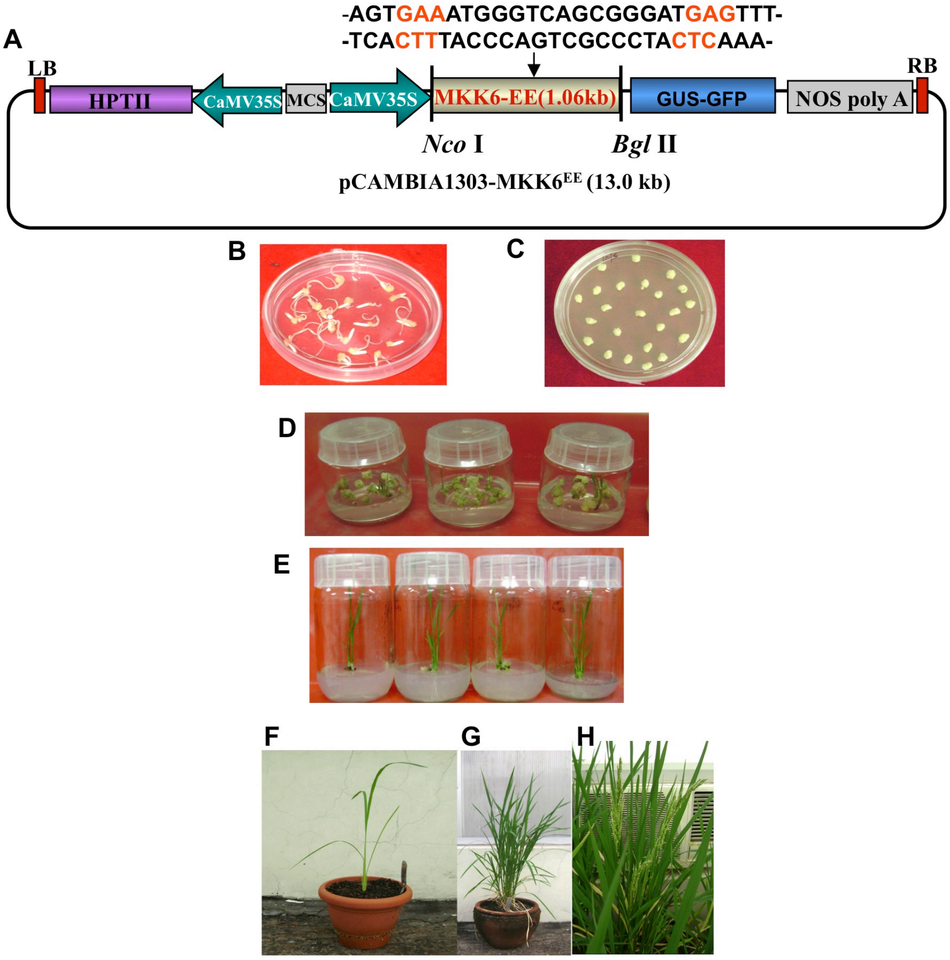


**Figure S2 *Agrobacterium* mediated transformation of rice (PB1).** (A) T-DNA in binary vector pCAMBIA 1303 containing full length *OsMKK6* with mutation at S221E and T227 at *Nco* I/*Bgl* II sites. (B) Callus emerges from rice seeds on MS medium supplemented with 2,4-D. (C) calli ready for *Agrobacterium* transformation. (D) regenerating calli on selection media containing 50mg/lt hygromycin. (E) Putative transgenic plants transferred to culture bottles on MS for rooting. (F) Rooted plants are transferred to pots in green house. (G) Mature plants after 3 months of growth. (H) Plants bearing panicles in green house after 5 months of regeneration.
