# Supplementary material for: Overexpression of constitutively active mitogen activated protein kinase kinase 6 enhances tolerance to salt stress in rice
Source: Rice (N Y). 2013 Oct 28;6:25. doi: 10.1186/1939-8433-6-25 (PMC4883705; doi:10.1186/1939-8433-6-25)
Supplement: Supplementary file 4 — Additional file 4: Figure S3: Confirmation of transgenic lines. (A) Testing of 39 putative transgenic lines for the presence of OsMKK6 transgene by total genomic PCR using flanking sequence of pCAMBIA primer, ‘C’ is the amplification of control (wild type) and M is DNA ladder (500 bp). (B) Northern blot analysis of 10 transgenic lines overexpressing OsMKK6EE lines. OsMKK6 cDNA was used as a radiolabeled probe for northern hybridization, C is wild type rice plants. The lower panel show methylene blue stained rRNA for equal loading and RNA quality. (PDF 375 KB) [file 12284_2013_76_MOESM4_ESM.pdf]

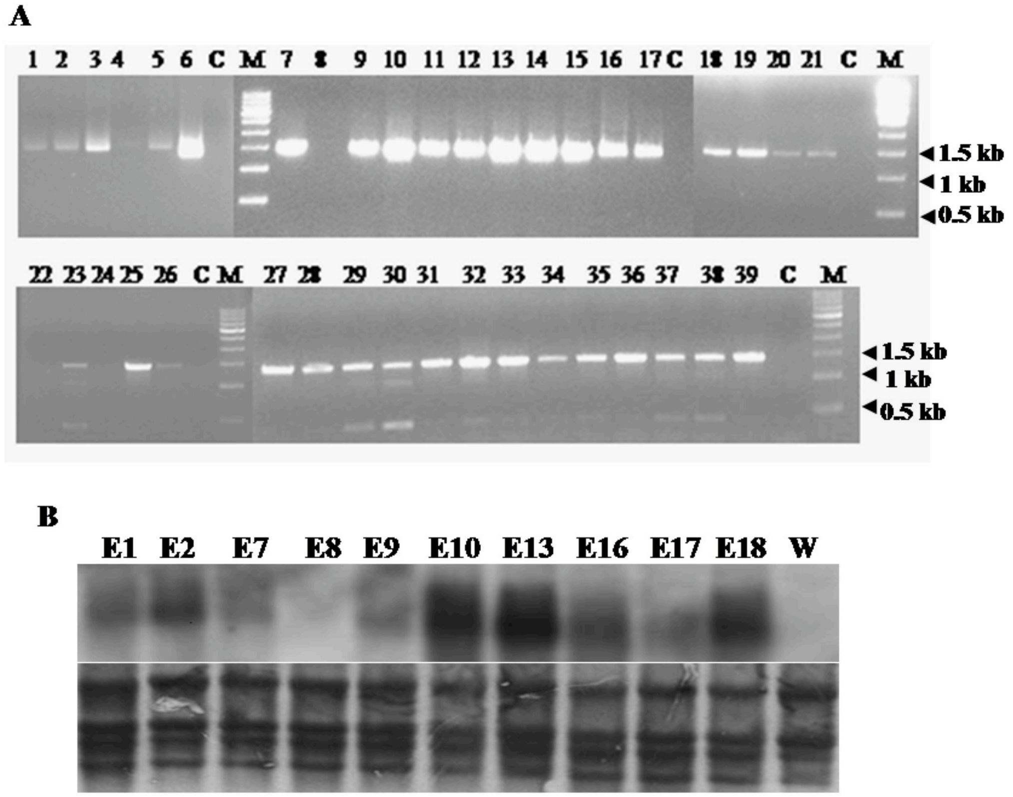

Figure S3 Confirmation of transgenic lines. (A) Testing of 39 putative transgenic lines for the presence of *OsMKK6* transgene by total genomic PCR using flanking sequence of pCAMBIA primer, 'C' is the amplification of control (wild type) and M is DNA ladder (500 bp). (B) Northern blot analysis of 10 transgenic lines overexpressing *OsMKK6*^{EE} lines. *OsMKK6* cDNA was used as a radiolabeled probe for northern hybridization, C is wild type rice plants. The lower panel show methylene blue stained rRNA for equal loading and RNA quality.
