# Supplementary material for: Overexpression of constitutively active mitogen activated protein kinase kinase 6 enhances tolerance to salt stress in rice
Source: Rice (N Y). 2013 Oct 28;6:25. doi: 10.1186/1939-8433-6-25 (PMC4883705; doi:10.1186/1939-8433-6-25)
Supplement: Supplementary file 5 — Additional file 5: Figure S4: Chlorophyll fluorescence of overexpressed lines. (A) Maximum quantum yield (Fv/Fm), (B) Effective quantum yield (Yield II) of overexpressed transgenic lines. SD from three different leaves with three points on one leaf of same line has been represented. No statistically significant difference was noticed with control as wild type 3 at P < 0.05 and P < 0.001. (PDF 333 KB) [file 12284_2013_76_MOESM5_ESM.pdf]

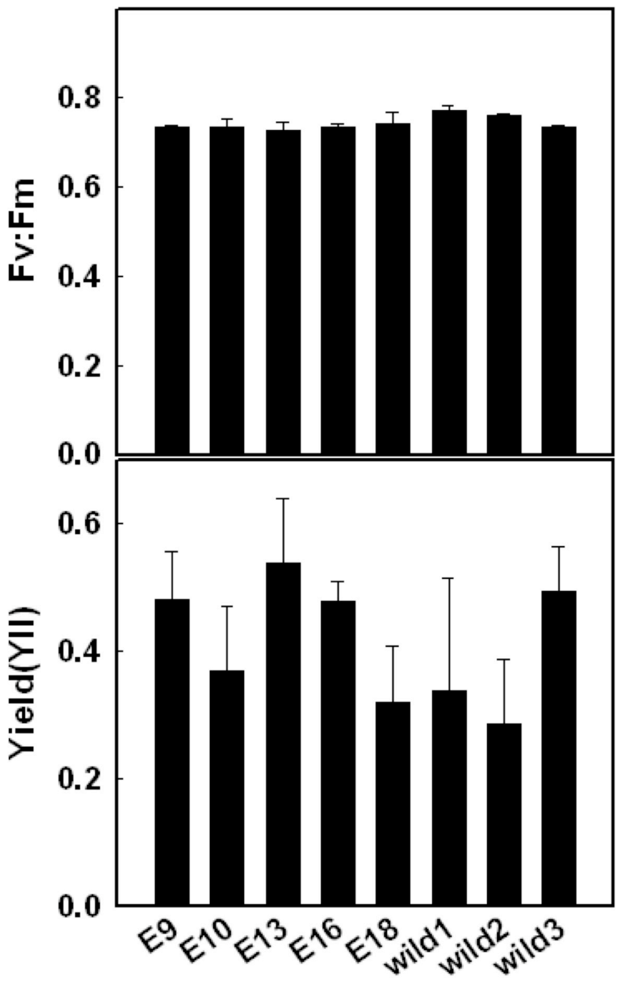

Figure S4 Chlorophyll fluorescence of overexpressed lines. (A) maximum quantum yield (Fv/Fm), (B) Effective quantum yield (Yield II) of overexpressed transgenic lines. SD from three different leaves with three points on one leaf of same line has been represented. No statistically significant difference was noticed with control as wild type 3 at $P<0.05$ and $P<0.001$.
