# Supplementary figures and images for: Overexpression of constitutively active mitogen activated protein kinase kinase 6 enhances tolerance to salt stress in rice
Source: Rice (N Y). 2013 Oct 28;6:25. doi: 10.1186/1939-8433-6-25 (PMC4883705; doi:10.1186/1939-8433-6-25)

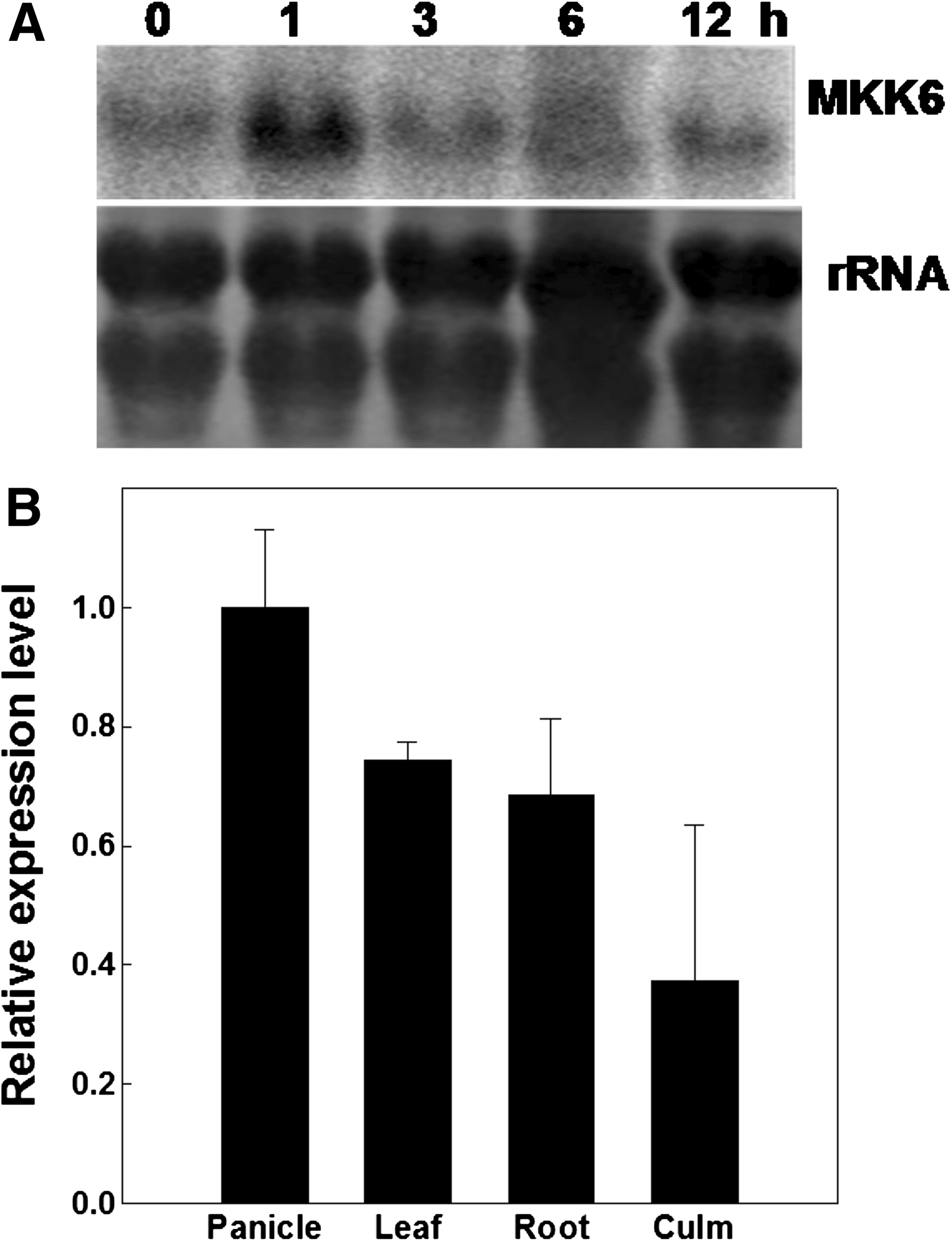

Supplement: Supplementary file 6 — Authors’ original file for figure 1 [file 12284_2013_76_MOESM6_ESM.tif]

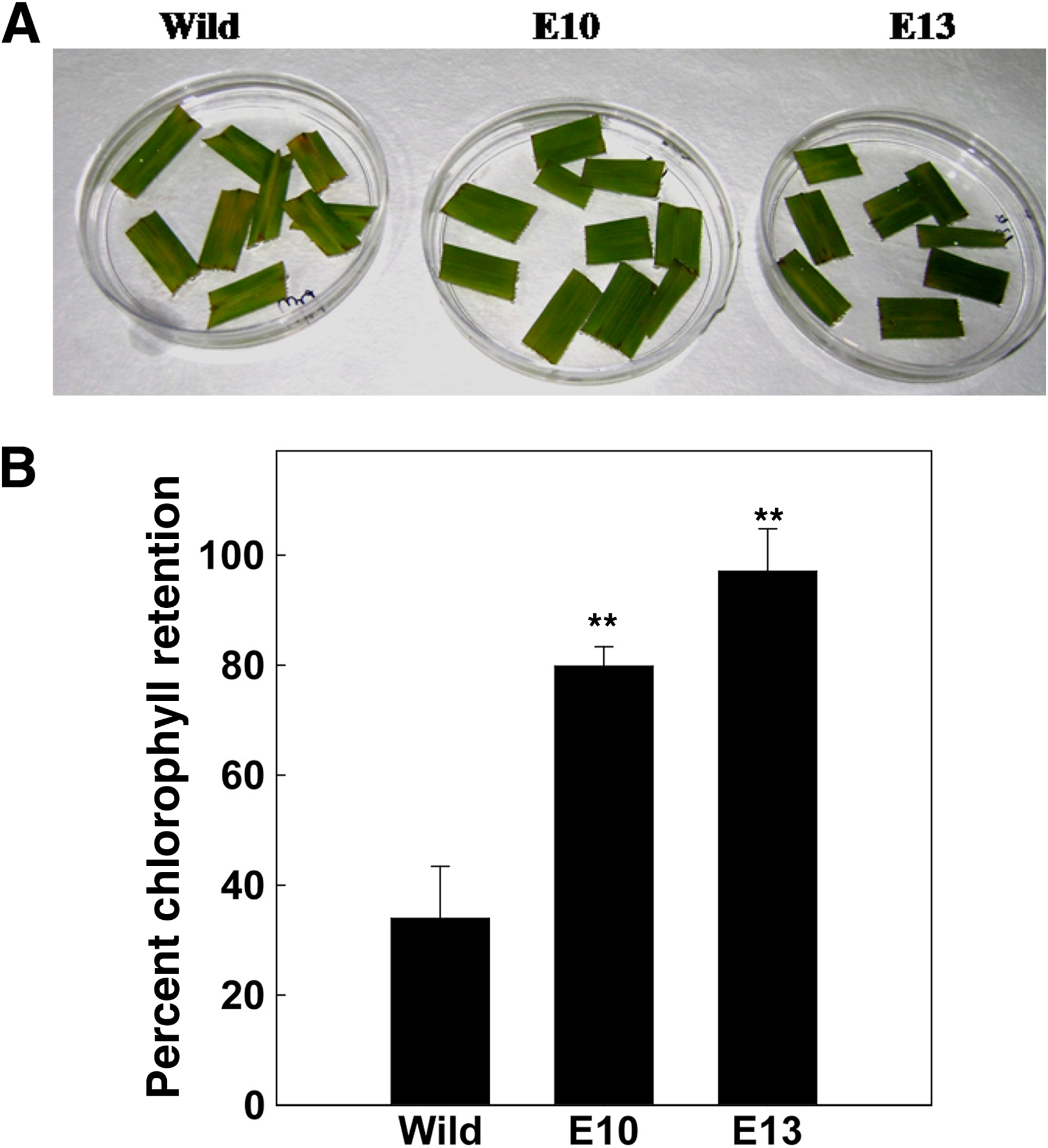

Supplement: Supplementary file 7 — Authors’ original file for figure 2 [file 12284_2013_76_MOESM7_ESM.tiff]

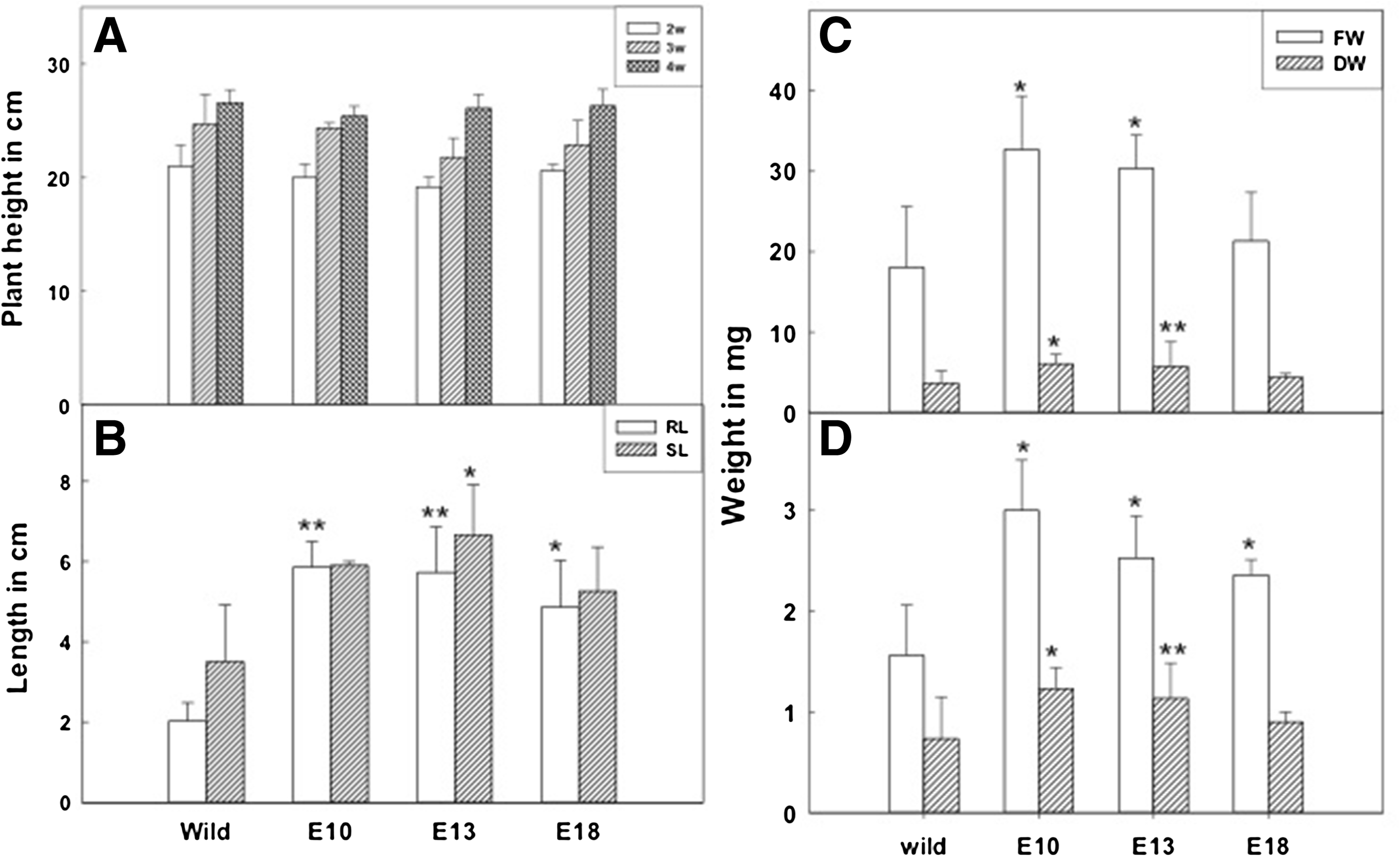

Supplement: Supplementary file 8 — Authors’ original file for figure 3 [file 12284_2013_76_MOESM8_ESM.tiff]

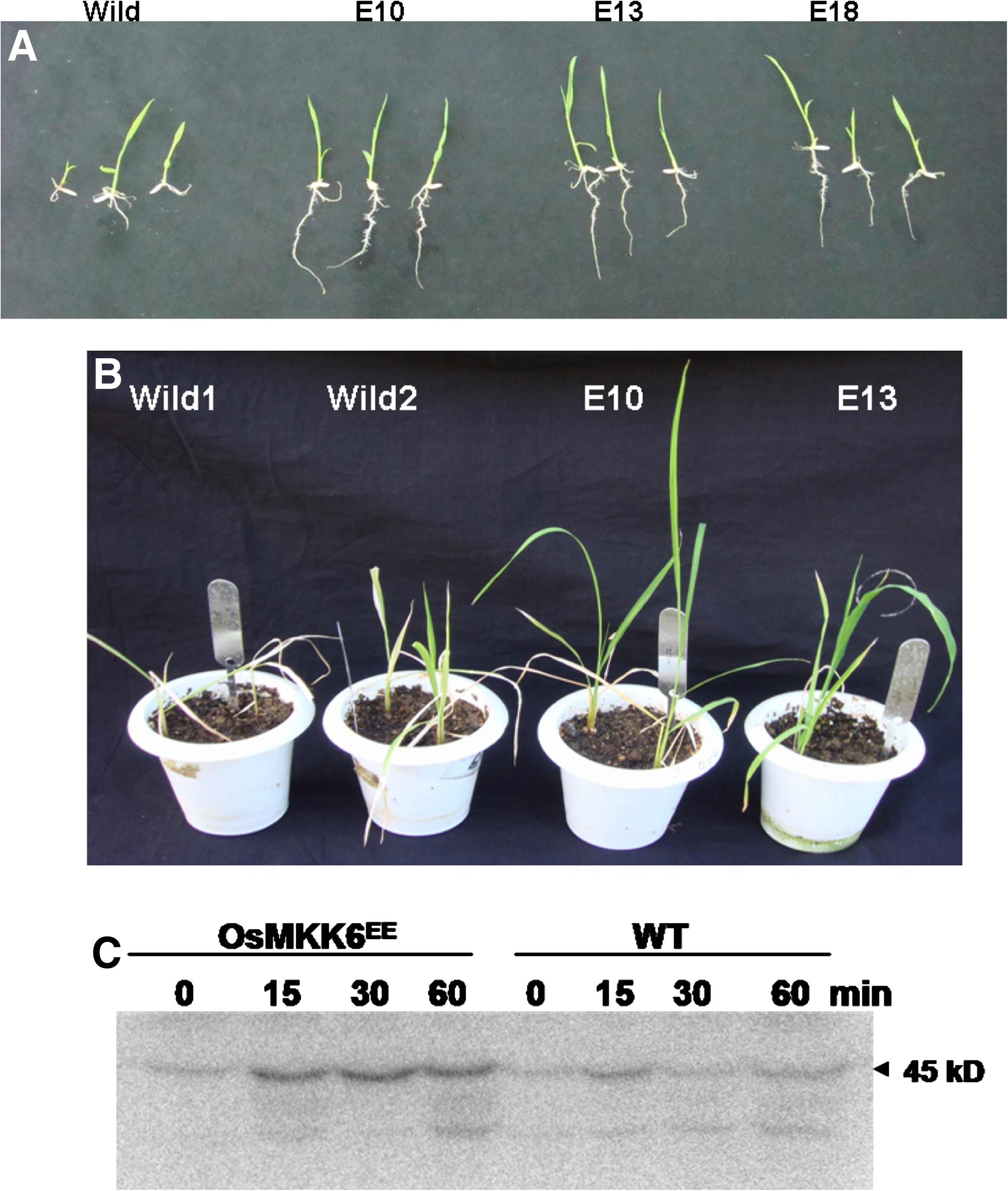

Supplement: Supplementary file 9 — Authors’ original file for figure 4 [file 12284_2013_76_MOESM9_ESM.tif]
